# Chloroplast Genome Analysis of Six *Camellia sinensis* Accessions: Genetic Divergence, Adaptive Evolution, and Molecular Marker Development

**DOI:** 10.3390/biology15010007

**Published:** 2025-12-19

**Authors:** Yanli Fu, Lei Pan, Xiaoxi Du, Zhigang Hao

**Affiliations:** 1 Key Laboratory of Integrated Pest Management on Crops in Northwestern Oasis, Ministry of Agriculture and Rural Affairs, National Plant Protection Scientific Observation and Experiment Station of Korla, Xinjiang Key Laboratory of Agricultural Biosafety, Institute of Plant Protection, Xinjiang Uygur Autonomous Region Academy of Agricultural Sciences, Urumqi 830091, China; 18701075950@163.com; 2 Hainan Seed Industry Laboratory, Sanya 572025, China; pp1181494002@163.com; 3 Hainan Key Laboratory for Biosafety Monitoring and Molecular Breeding in Off-Season Reproduction Regions, State Key Laboratory of Tropical Crop Breeding, Institute of Tropical Bioscience and Biotechnology & Sanya Research Institute, Chinese Academy of Tropical Agricultural Sciences, Sanya 572025, China; duxiaoxi@catasitbb.cn

**Keywords:** *Camellia sinensis* ‘hainanensis’, chloroplast genome, phylogenetic analysis, adaptive evolution, molecular marker development

## Abstract

*Camellia sinensis* ‘hainanensis’ (Hainan Sheng tea) is an endemic tea germplasm resource native to Hainan Island, China. Using complete chloroplast genome sequencing combined with comprehensive comparative analyses, we elucidated the genetic architecture of six *C. sinensis* accessions. The chloroplast genomes exhibited a typical quadripartite circular structure (~157 Kb) comprising 80 unique protein-coding genes, 30 tRNA genes, and 4 rRNA genes. Expansion and contraction of the inverted repeat (IR) regions led to boundary shifts affecting genes, while nucleotide diversity within the large single-copy (LSC) and small single-copy (SSC) regions (Pi > 0.0035) markedly exceeded that of the conserved IR regions. Phylogenetic reconstruction revealed that *C. sinensis* ‘hainanensis’ shared the closest evolutionary relationship with Yunnan large-leaf tea (*Camellia grandibracteata*), supporting its independent lineage within the genus. A polymorphic molecular marker derived from the hypervariable non-coding region (*trnT–psbD*) may serve as a useful preliminary marker for distinguishing *C. sinensis* ‘hainanensis’ from related taxa and hybrids. This study provides the first comprehensive comparison of complete chloroplast genomes of six *C. sinensis* ‘hainanensis’, identifies three distinct plastome types, and develops a molecular marker that can reliably distinguish these types, offering valuable genomic resources for future studies on tea evolution and germplasm identification.

## Simple Summary

Scientists studied a special type of tea plant that is native to Hainan Island in China. Their aim was to understand its unique genetic blueprint in order to protect and make better use of this valuable plant. By analyzing the complete chloroplast genome, they compared six different tea accessions. They found that it could be grouped into three distinct genetic types. The researchers also discovered a tiny, specific difference in the DNA of one variety that acts like a unique fingerprint. This DNA fingerprint can be used to quickly and accurately distinguish this variety from others. This work provides a deeper understanding of the evolution of Hainan tea and offers a simple genetic tool for identifying it, which will be important for conserving this special variety and breeding improved plants in the future.

## 1. Introduction

*C. sinensis* ‘hainanensis’, an endemic tea germplasm native to Hainan Island, China, has recently garnered considerable attention owing to its unique genetic background and remarkable ecological adaptability [1,2]. Genomic, morphological, and phytochemical analyses have confirmed *C. sinensis* ‘hainanensis’ as a distinct species within the *Camellia* genus, exhibiting independent evolutionary characteristics [3]. Its primary distribution occurs in Wuzhishan City, located in the central–southern interior of Hainan Island (18.78° N, 109.52° E). Wuzhishan’s warm, humid climate and high-elevation mountainous environment provides suitable conditions for the growth of wild tea plants, which are mainly distributed above 500 m, with the largest population in Shuimen Township [4,5]. These environmental conditions collectively contribute to the exceptional quality and biochemical distinctiveness of *C. sinensis* ‘hainanensis’ (The Official Website of the Wuzhishan Municipal People’s Government, wzs.hainan.gov.cn, accessed 10 October 2022). Despite its designation as a national tea cultivar [6], *C. sinensis* ‘hainanensis’ retains wild genetic traits, including high genetic diversity and strong stress tolerance. Nevertheless, the lack of comprehensive germplasm surveys and systematic breeding efforts continues to limit its agricultural development and industrial utilization.

Organellar genomes (the chloroplast and mitochondrial genomes) represent essential components of plant genetic systems, both originating from ancient endosymbiotic events during the early evolution of eukaryotes [7]. In angiosperms, chloroplast genomes (cpDNA) typically possess a conserved circular quadripartite structure of approximately 120–160 Kb, encoding 110–130 genes that participate primarily in photosynthesis, transcription, and translation [8]. Owing to their uniparental (predominantly maternal) inheritance, low recombination rates, and conserved gene organization, chloroplast genomes serve as powerful molecular tools for phylogenetic inference, species identification, and population genetic analyses.

Recent studies have demonstrated the utility of comparative chloroplast genomics in developing lineage-specific molecular markers. For example, comparative analysis of the chloroplast genomes of four artificial *Taxodium* hybrids and their parental species enabled the identification of novel molecular markers [9]. Similarly, chloroplast genome comparisons among three *Paraphalaenopsis* species (*P. labukensis*, *P. deneveli*, and *P. laycockii* ‘Semi-alba’) revealed six hypervariable regions suitable for DNA marker development [10]. Moreover, comparative analyses of Saccharum spp. chloroplast genomes have shown the feasibility of using chloroplast-derived markers to discriminate closely related Saccharum species and their relatives [11]. Building upon these insights, the present study aims to sequence and compare the complete chloroplast genomes of *C. sinensis* ‘hainanensis’ and its related species, with the goal of identifying lineage-specific markers and providing a theoretical foundation for the conservation and sustainable utilization of this rare germplasm resource.

Given the evolutionary and functional importance of chloroplast genomes, exploring the cpDNA of *C. sinensis* ‘hainanensis’ provides an opportunity to unravel its adaptive evolution and guide conservation efforts. *C. sinensis* ‘hainanensis’ possesses remarkable scientific and socioeconomic significance as a distinctive tropical tea germplasm. First, its geographic isolation and unique evolutionary trajectory make it an ideal “natural laboratory” for investigating tea adaptation and speciation processes. The tropical-edge position of Hainan Island, combined with its diverse microclimatic conditions—particularly the high-altitude cloud forest zones—has fostered the development of genomic, metabolic, and stress resilience traits that differ markedly from those of other tea varieties. Nevertheless, systematic investigations into the genetic diversity and adaptive mechanisms of *C. sinensis* ‘hainanensis’ remain limited, constraining both its conservation and potential utilization.

Second, wild populations of *C. sinensis* ‘hainanensis’ are increasingly threatened by habitat fragmentation resulting from rainforest exploitation, climate change, and human disturbances. Many ancient trees now face the risk of extinction. Genomic-level insights into their population structure, genetic variation, and adaptive genes are urgently needed to inform effective conservation and restoration strategies.

Third, the economic potential of *C. sinensis* ‘hainanensis’ remains largely untapped. Its leaves contain elevated levels of polyphenols, amino acids, and distinctive tropical floral–fruity flavor compounds compared with small-leaf tea cultivars; however, the molecular mechanisms underlying these traits are still poorly understood. Integrating chloroplast and nuclear genomic analyses offers an opportunity to elucidate the regulatory networks involved in the biosynthesis of flavor-related metabolites, thereby supporting targeted quality improvement and molecular breeding.

Previous studies on *Camellia* chloroplast genomes have mainly focused on cultivated tea from mainland China [12], and no comprehensive cp–genome comparison has been conducted for Hainan tea resources. Moreover, plastome structural variation within Hainan *C. sinensis* has never been investigated, and no molecular markers are available to distinguish its plastome types. To fill these gaps, we assembled and characterized the complete chloroplast genomes of six *C. sinensis* ‘Hainanensis’ accessions, compared their genome structures and IR boundaries, identified hypervariable regions, inferred their phylogenetic relationships within the *Camellia* genus, and developed a molecular marker capable of distinguishing the plastome types.

## 2. Materials and Methods

### 2.1. Genome Sequencing, Assembly, and Annotation

Plant material for each accession was collected from a single healthy individual in Shuimanxiang, Wuzhishan City, Hainan Province, China (18.88° N, 109.66° E) on 4 March 2024. Genomic DNA was extracted from leaves using CTAB extraction method. and used to construct 350 bp paired-end sequencing libraries, which were sequenced on the Illumina NovaSeq 6000 platform (San Diego, CA, USA). Raw reads were assembled into complete circular chloroplast genomes using GetOrganelle (v1.7.5) (parameter: -t 20 -k 87,107,117,127 -F embplant_pt) [13]. Genome annotation was conducted using CPGAVAS2 (http://47.96.249.172:16019/analyzer/home, accessed on 30 November 2025) [14], and annotation accuracy was manually verified and corrected in Apollo (v3.0) [15]. Transfer RNA (tRNA) and ribosomal RNA (rRNA) genes were predicted using tRNAscan-SE (v2.0) [16], and BLASTn [17], respectively. Circular chloroplast genome maps were visualized using OGDRAW (v1.3.1) [18].

### 2.2. IR Boundary and Codon Usage Bias Analysis

The junctions between the LSC, SSC, and IR regions (i.e., LSC–IRb, IRb–SSC, SSC–IRa, and IRa–LSC) were analyzed and graphically represented with IRscope (https://irscope.shinyapps.io/irapp/, accessed on 30 November 2025) [19]. PCGs from three types of *Camellia* species were extracted by PhyloSuite (v1.2.2) [20]. Codon usage bias was analyzed in MEGA (v7.0) [21]. Relative synonymous codon usage (RSCU) values were calculated to assess codon preference patterns across all chloroplast genomes.

### 2.3. Repeat Sequence Analysis

Simple sequence repeats (SSRs) were detected using MISA with parameter: 1-8, 2-4, 3-4, 4-3, 5-3, and 6-3, where 1, 2, 3, 4, 5, and 6 indicate the monodi-, tri-, tetra-, penta-, and hexa-nucleotide repeats [22]. Tandem repeats were identified using Tandem Repeats Finder (TRF) (parameter: default), while dispersed repeats—including palindromic, forward, reverse, and complementary types—were analyzed using the REPuter web server (parameter: minimal size 30 bp, hamming distance 3) [23]. Data visualization and comparative plots were generated in Microsoft Excel 2021 and Circos (http://circos.ca/, accessed on 30 November 2025) [24].

### 2.4. Phylogenetic Analysis

The 48 *Camellia* species were selected to represent major lineages across the genus. First, eighty shared PCGs from 48 *Camellia* species were extracted using PhyloSuite (v1.2.2) [20], then aligned using MAFFT (v7) [25]. A maximum-likelihood (ML) tree was reconstructed in IQ-TREE (v1.6.12) [26] using the best-fit model identified by ModelFinder. For the PCGs dataset, the model K3Pu+F+I+G4 was applied, while for the whole-plastome dataset, K3Pu+F+I was used. In both analyses, branch support was assessed with the parameters: --alrt 1000 -B 1000. All resulting trees were visualized using iTOL v7 (https://itol.embl.de/, accessed on 30 November 2025) [27].

### 2.5. Nucleotide Diversity, Sequence Similarity, and Synteny Analysis

Whole chloroplast genome alignments were generated using MAFFT (v7) [25]. Nucleotide diversity (Pi) was calculated in DnaSP (v6.0) [28] with a sliding window of 500 bp and a step size of 100 bp. Sequence similarity among chloroplast genomes was further evaluated using mVISTA (http://genome.lbl.gov/vista/index.shtml, accessed on 30 November 2025) in Shuffle-LAGAN mode [29]. Whole-genome alignments were performed in Mauve (http://darlinglab.org/mauve/mauve.html, accessed on 30 November 2025) [30] to detect potential structural rearrangements and assess overall genomic synteny among the six chloroplast genomes.

### 2.6. Molecular Marker Development and Validation

Primers targeting the hypervariable *trnT–psbD* intergenic spacer were designed using NCBI Primer-BLAST (https://www.ncbi.nlm.nih.gov/tools/primer-blast/, accessed on 30 November 2025). Primer synthesis was carried out by Boshang Biotechnology Co., Ltd., Lianyungang, China Primer sequences are listed in Table S9. Polymerase chain reaction (PCR) was performed in a 20 µL reaction mixture containing 10 µL of 10× reaction buffer, 5 pmol of each primer, 1.25 U of Taq DNA polymerase, and 20 ng of genomic DNA. The amplification protocol consisted of an initial denaturation at 94 °C for 5 min, followed by 35 cycles of 94 °C for 30 s, 56–62 °C for 30 s, and 72 °C for 30 s, with a final extension at 72 °C for 7 min. PCR amplification products were separated by agarose gel electrophoresis, purified, and verified through Sanger sequencing.

To validate the marker, a total of six individuals representing three groups—Type 1 (‘Anjibaicha’ ‘Fuding dahaocha’ ‘Fuyun 6’ and ‘Zhongcha 108’), Type 2 (‘Fuding dabaicha’), and Type 3 (‘Dayecha’)—were analyzed. This design allows assessment of the robustness and specificity of the four-thymine (T_4_) insertion identified in ‘Dayecha’.

## 3. Results

### 3.1. Chloroplast Genome Assembly and Annotation

**Figure 1:**
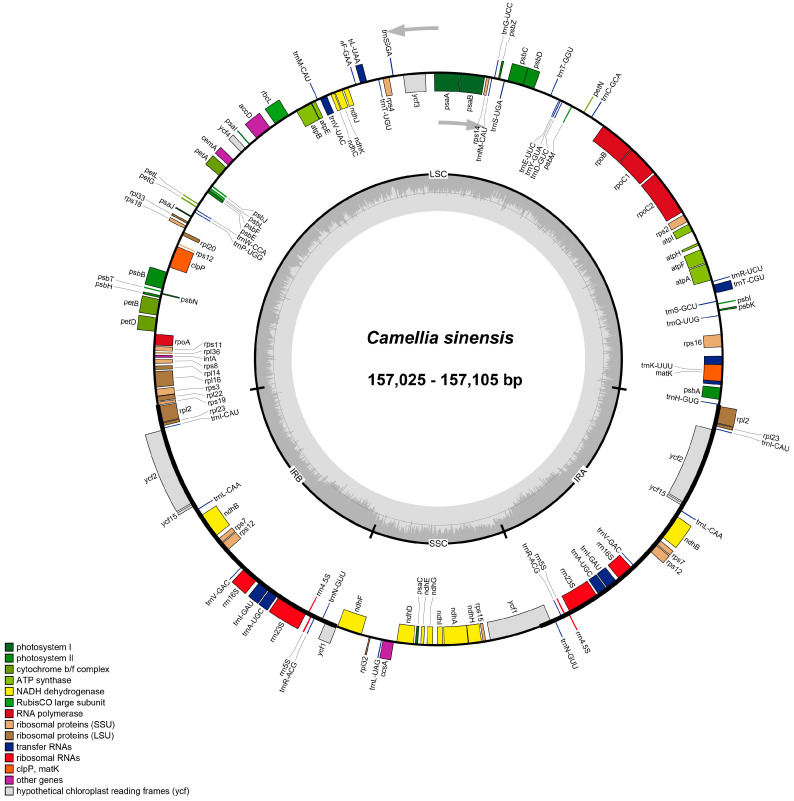
Circular maps of chloroplast genomes of six *C. sinensis* cultivars. Each map illustrates the typical quadripartite structure of the chloroplast genome, consisting of a large single-copy (LSC) region, a small single-copy (SSC) region, and two inverted repeat (IRa and IRb) regions. Genes are color-coded according to their functional categories, including photosystem components, cytochrome b/f complex, ATP synthase, NADH dehydrogenase, ribosomal proteins, RNA polymerases, ribosomal RNAs, transfer RNAs, and hypothetical reading frames (*ycf*). The inner circle indicates the GC content variation across the genome, while arrows denote the transcriptional direction of each gene.

Sequencing was performed on the Illumina NovaSeq platform with 150 bp paired-end reads. After quality filtering (Q30 > 95%, minimum read length = 50 bp), clean data for each accession ranged from 10.05 to 19.47 Gb, resulting in an average chloroplast genome coverage of 535.65×~1919.23× (Table S1, Figure S1).The chloroplast genomes of six *C. sinensis* ‘hainanensis’ cultivars were successfully assembled, each exhibiting the typical circular quadripartite structure characteristic of angiosperms, comprising a large single-copy (LSC) region, a small single-copy (SSC) region, and two inverted repeat (IR) regions (Figure 1). The total genome sizes ranged from 157,025 bp to 157,105 bp, with an average GC content of approximately 37.3%. According to the differences in the lengths of the LSC, SSC, and IR regions among the six cultivars, six *C. sinensis* ‘hainanensis’ cultivars categorized into three types: Type 1 (‘Anjibaicha’ ‘Fuding dahaocha’ ‘Fuyun 6hao’ and ‘Zhongcha 108’), Type 2 (‘Fuding dabaicha’), and Type 3 (‘Dayecha’) (Table 1). Genome annotation identified 80 unique protein-coding genes (PCGs), 30 transfer RNA (tRNA) genes, and 4 ribosomal RNA (rRNA) genes (Table 2). Among these, eight PCGs and seven tRNAs occurred in duplicate, reflecting the conserved yet subtly variable organization typical of *Camellia* chloroplast genomes.

### 3.2. IR Boundary Dynamics

**Figure 2:**
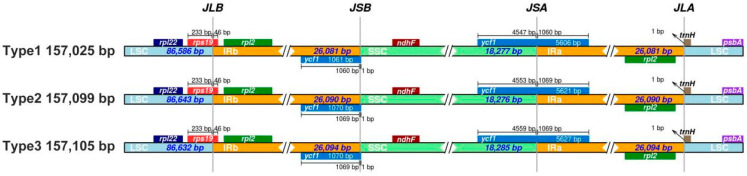
Comparison of inverted repeat (IR) boundary regions among three *Camellia* chloroplast genomes. Schematic representation of the junctions between single-copy regions (LSC and SSC) and inverted repeat regions (IRa and IRb) in the three *Camellia* chloroplast genome types. Gene positions and boundary distances (in bp) are shown relative to each junction. JLB, JSB, JSA, and JLA denote the junction points between LSC/IRb, IRb/SSC, SSC/IRa, and IRa/LSC regions, respectively. The comparison highlights structural variations and IR boundary shifts among the chloroplast genomes.

Comparative analysis of the junctions between the IR and single-copy (SC) regions revealed clear structural differences among the three types of *C. sinensis* ‘Hainanensis’ chloroplast genomes. Boundary-associated genes, such as ycf1, were located at or near the IR/SC junctions with type-specific distances. For example, in Type 1, *ycf1* was positioned 1061 bp from the IRb/SSC boundary, whereas in Type 2 and Type 3, the distance was 1070 bp. At the SSC/IRa junction, the length of the *ycf1* gene extending into the IRa region also differed, measuring 4547 bp in Type 1, 4553 bp in Type 2, and 4559 bp in Type 3. These observations described structural variation among plastome types and were consistent with established models of chloroplast genome evolution in angiosperms (Figure 2, Table S2).

### 3.3. Codon Usage Bias Analysis

**Figure 3:**
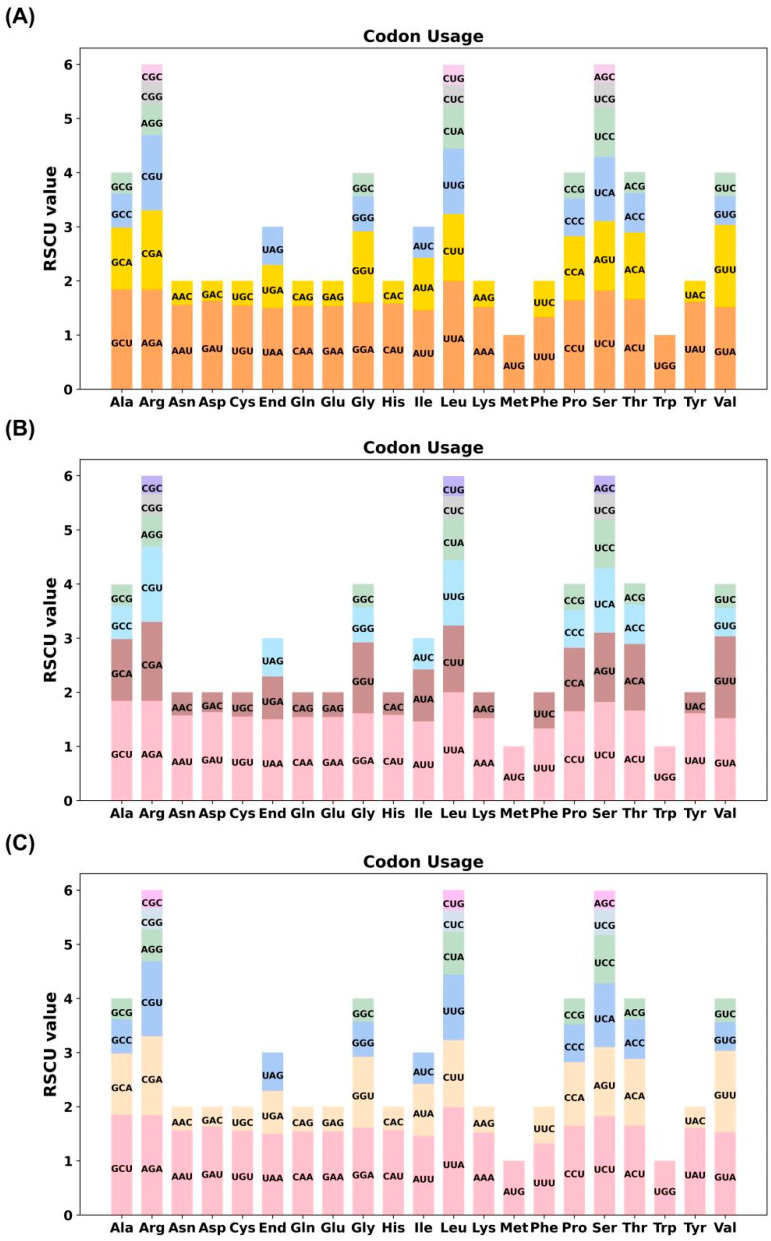
Relative synonymous codon usage (RSCU) patterns in the chloroplast genomes of three types of *C. sinensis* ‘hainanensis’. (**A**–**C**) represent the RSCU values of PCGs for Type1, Type2, and Type3 of *C. sinensis* ‘hainanensis’, respectively. The x-axis indicates amino acids and their corresponding synonymous codons, while the y-axis shows the RSCU values. Codons with RSCU > 1.0 are used more frequently than expected, indicating codon usage bias, whereas codons with RSCU < 1.0 are used less frequently. Different colors denote distinct codons associated with each amino acid. The comparative RSCU profiles reveal conserved yet species-specific codon preference patterns among the analyzed *Camellia* chloroplast genomes.

Codon usage bias was evaluated by calculating the relative synonymous codon usage (RSCU) values for all PCGs across the three types of chloroplast genomes. With the exception of the start codon AUG (methionine) and tryptophan (Trp; UGG), which exhibited no bias (RSCU = 1), most amino acids displayed distinct preferences for specific synonymous codons. Among them, leucine (Leu) showed a pronounced bias toward the UUA codon in all genomes, with RSCU values reaching up to 2.0 (Figure 3; Table S3). Across the three identified chloroplast genome types, codon usage patterns remained highly conserved. An RSCU value greater than 1 reflects a positive codon usage bias, indicating preferential use of certain codons beyond random expectation. The observed consistency in codon preference among the six cultivars suggests a conserved pattern of codon usage in *Camellia* chloroplast genomes.

### 3.4. Repeat Sequence Analysis

**Figure 4:**
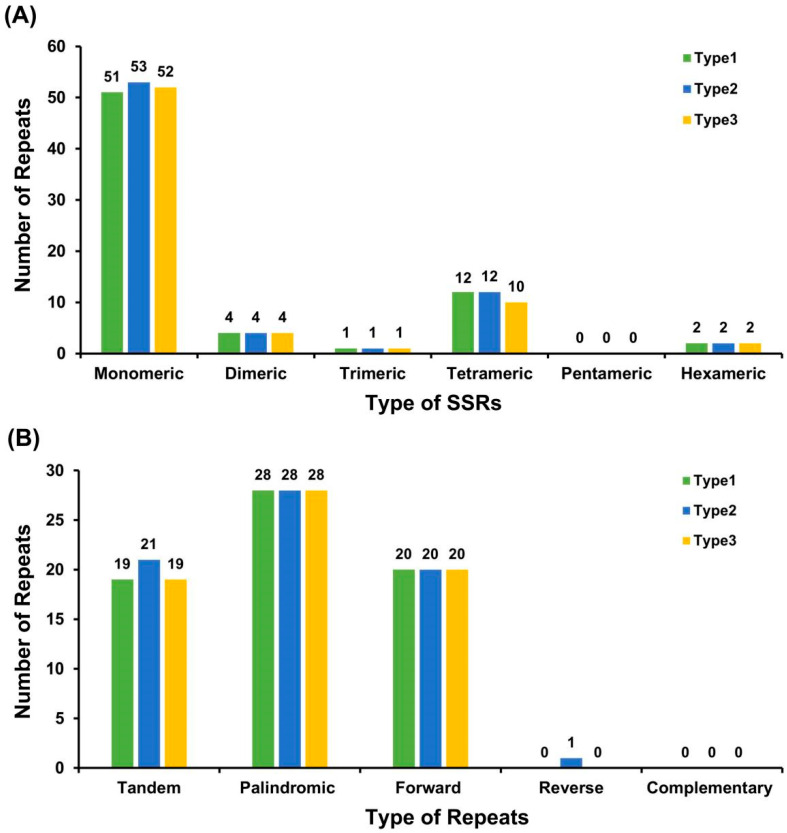
Analysis of repetitive sequences in the chloroplast genomes of *C. sinensis* ‘hainanensis’ three types. (**A**) Distribution of simple sequence repeats (SSRs) classified by motif type (monomeric, dimeric, trimeric, tetrameric, pentameric, and hexameric) among three *Camellia* chloroplast genomes. (**B**) Distribution of dispersed repeats (≥30 bp), categorized by repeat type: tandem, palindromic, forward, reverse, and complementary. Different colors represent the three analyzed *Camellia* chloroplast genomes (Type1, Type2, and Type3). The overall SSR and repeat patterns are highly conserved among species, with mononucleotide SSRs and palindromic repeats being the most abundant categories.

A total of 69–72 simple sequence repeats (SSRs) were identified across the three plastome types of *C. sinensis* ‘hainanensis’. Mononucleotide and dinucleotide repeats were predominant, accounting for 78.57–81.16% of all SSRs. Among mononucleotide motifs, thymine (T) repeats were the most abundant (52.94–57.69%). Although the overall SSR composition was conserved among Type1, Type2, and Type3, slight quantitative differences were observed. Type1–Type3 contained 51, 53, and 52 mononucleotide SSRs, respectively, while tetranucleotide SSRs ranged from 10 to 12. Trinucleotide and dinucleotide SSRs were present at identical numbers in all plastomes, and no pentanucleotide SSRs were detected (Figure 4A; Table S4).

In addition to SSRs, dispersed repeats—including palindromic, forward, tandem, and a minor proportion of reverse repeats (≥30 bp)—were also detected. Each plastome harbored 48–49 repeat pairs, with palindromic repeats being the most frequent type (28 in each plastome). Tandem repeats varied slightly (19–21), whereas forward repeats were identical across types (20). A single reverse repeat was detected only in Type2, and complementary repeats were absent from all plastomes. The longest forward repeat measured 82 bp. These repetitive elements may contribute to chloroplast genome stability and potentially mediate structural rearrangements (Figure 4B; Tables S5 and S6).

### 3.5. Phylogenetic Analysis

**Figure 5:**
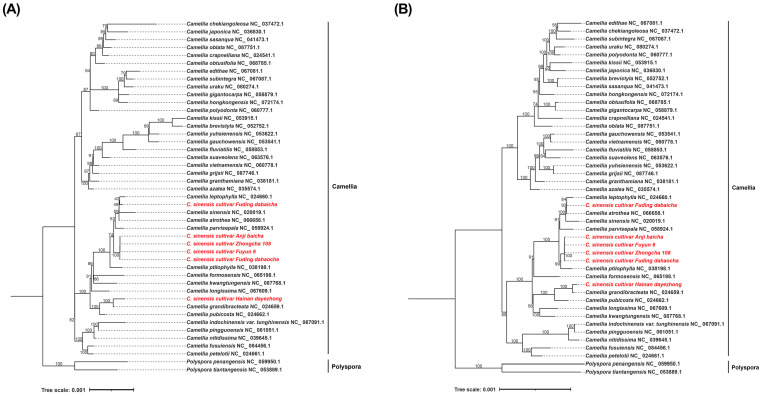
Phylogenetic relationships among *Camellia* species. (**A**) Maximum-likelihood (ML) phylogenetic tree constructed from complete chloroplast genome sequences of the analyzed *Camellia* species. (**B**) ML phylogenetic tree based on concatenated shared PCGs across the same species. The maximum-likelihood (ML) phylogenetic tree was reconstructed using concatenated chloroplast PCGs. Bootstrap support values (≥50) are indicated at the corresponding nodes. The analysis includes multiple *Camellia* species, with *Aspidistra crassifolia* and *A. dolichanthera* designated as outgroups. Accession numbers and species names used in the phylogenetic reconstruction are listed in Table S7. The red-highlighted taxa represent the species analyzed in this study. note: fuding dahaocha—dahaocha, dayecha—hainan dayezhong, fuyun6—fuyun 6 hao.

A maximum-likelihood (ML) phylogenetic tree was reconstructed using both the complete plastome sequences and the 80 shared PCGs from 48 *Camellia* species to resolve the evolutionary relationships among the tea cultivars. Phylogenetic analyses based on both the complete plastome sequences (Figure 5A) and the 80 shared PCGs (Figure 5B) yielded highly congruent topologies. Four cultivars classified as Type1 (‘Anjibaicha’, ‘Dahaocha’, ‘Fuyun 6hao’, and ‘Zhongcha 108’) formed a well-supported clade that was sister to *C. ptilophylla*, indicating a close evolutionary relationship. Type2 (‘Fuding Dabaicha’) showed strong affinity to *C. grandibracteata* and *C. pubicosta*, whereas Type3 (‘Dayecha’) was most closely related to *C. leptophylla*. These phylogenetic patterns highlight the genetic differentiation among the three plastome types and provide new insight into the domestication history of Hainan tea germplasm. Most nodes in the ML tree exhibited high bootstrap support (≥87), underscoring the robustness of the inferred relationships.

### 3.6. Nucleotide Diversity Analysis

**Figure 6:**
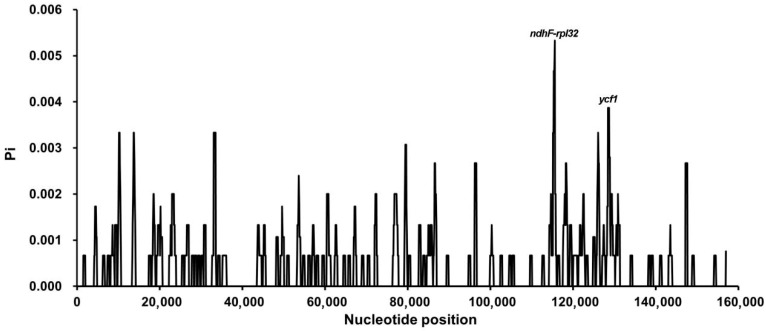
Nucleotide diversity (Pi) across the chloroplast genomes of three *Camellia* types. Sliding window analysis (window length = 500 bp; step size = 100 bp) was performed to estimate nucleotide diversity (Pi) among the chloroplast genomes. Peaks in the Pi curve indicate hypervariable regions, with the highest variability detected in the *ndhF–rpl32* intergenic spacer and the *ycf1* gene region.

**Figure 7:**
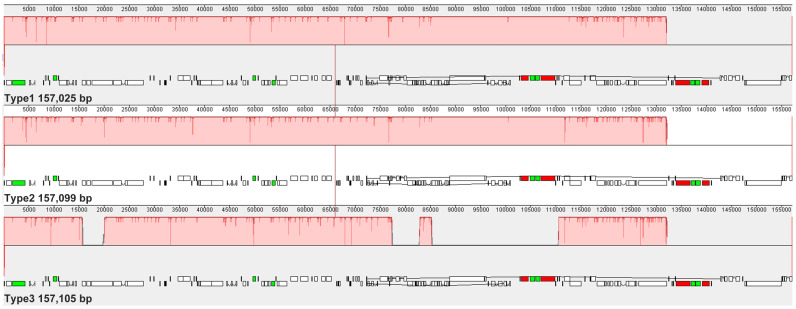
Synteny analysis of the three chloroplast genome types of *Camellia*. Synteny maps show locally collinear blocks (LCBs) among the three chloroplast genomes. Colored ribbons connect homologous regions, with red ribbons indicating inversions. The high degree of synteny and the absence of large-scale rearrangements suggest that the overall chloroplast genome structure is highly conserved among the analyzed *Camellia* types.

Nucleotide diversity (Pi) analysis revealed two hypervariable regions, *ndhF*–*rpl32* and *ycf1*, with Pi values exceeding the genome-wide average of 0.0035 (Table S8). These regions represent promising candidate loci for molecular marker development and species discrimination. Consistent with previous findings in *Camellia*, the LSC and SSC regions exhibited greater nucleotide variability than the IR regions (Figure 6). Alignment using mVISTA further confirmed high sequence similarity among the six chloroplast genomes, with the greatest conservation observed in the IR regions (Figure S2). These results underscore the overall structural stability of tea chloroplast genomes despite subtle sequence polymorphisms. Whole-genome alignment performed with Mauve revealed no major rearrangements among the six genomes. The high degree of collinearity observed indicates strong evolutionary conservation and functional constraint within the chloroplast genomes of tea plants (Figure 7).

### 3.7. Molecular Marker Development

**Figure 8:**
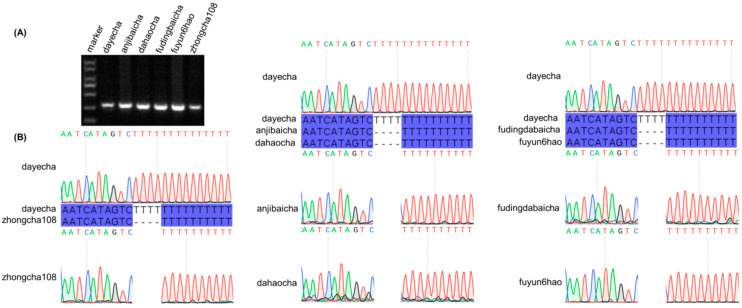
Validation of species-specific molecular markers in *Camellia* cultivars. (**A**) Agarose gel electrophoresis of PCR products amplified from the hypervariable chloroplast intergenic spacer *trnT–psbD*. Lane M: DL2000 DNA marker. (**B**) Sanger sequencing chromatograms confirming a four-thymine (T) insertion unique to the ‘dayecha’ cultivar. Sequence alignments among different *Camellia* accessions illustrate the single-nucleotide variation responsible for cultivar-specific differentiation.

The hypervariable non-coding region *trnT–psbD* was selected as the target locus for molecular marker development. A four-thymine (T_4_) insertion specific to the Type 3 cultivar ‘Dayecha’ (*C. sinensis* ‘hainanensis’) was identified as a polymorphic marker. The PCR amplification produced fragments of the expected size (Figure 8A), and subsequent Sanger sequencing of the amplified products confirmed the presence of this insertion, verifying the reliability of the designed molecular marker (Figure 8B; Table S9).

## 4. Discussion

In this study, the complete chloroplast genomes of six *C. sinensis* ‘hainanensis’ cultivars were assembled and annotated. The chloroplast genomes of *C. sinensis* ‘hainanensis’ and its related taxa from Hainan Province exhibited the typical circular quadripartite structure of angiosperms, containing 80 unique PCGs and 30 tRNA genes. The overall genomic architecture, gene content, and organization were highly conserved, consistent with the general pattern observed in other *Camellia* chloroplast genomes [12,31]. Despite this structural conservation, subtle variations were detected at the inverted repeat/single-copy (IR/SC) junctions and in total genome size among the three identified genome types (Type1–Type3). These shifts, particularly involving *ycf1* and *ndhF*, are common features of chloroplast genome dynamics previously reported in other *Camellia* species [8]. Such structural variations may influence gene copy number and overall genome stability, although their functional consequences in tea plants remain to be elucidated. These findings provide strong evidence for the highly conserved and slow evolution of the chloroplast genome in this genus.

Codon usage bias analysis revealed a pronounced preference for specific synonymous codons, particularly UUA for leucine, across all three types of chloroplast genomes. This result indicates a consistent codon usage pattern among the analyzed chloroplast genomes based on RSCU values. Furthermore, the abundance of mononucleotide and dinucleotide SSRs—especially poly-T motifs—and dispersed repeats suggests potential roles in chloroplast genome plasticity. These repeat elements represent valuable molecular resources for population genetics, phylogeography, and cultivar identification in *C. sinensis* ‘hainanensis’ and related taxa.

Tea quality traits such as flavor, aroma, and color are largely determined by metabolites including catechins, caffeine, and theanine, which vary among *Camellia* cultivars [32]. Selection during tea domestication has shaped genes involved in caffeine accumulation and lipid metabolism, while transcription factors such as MYB36, bHLH62, and NF-YB regulate catechin and theanine biosynthesis [33,34]. Together, these studies elucidate molecular mechanisms underlying key agronomic and flavor-related traits in tea. Our results reveal chloroplast genetic divergence among the analyzed cultivars. While these variations primarily reflect plastome differences, they could potentially influence photosynthesis, lipid metabolism, or secondary metabolite biosynthesis, which may indirectly contribute to flavor and ecological adaptation [35,36,37]. As nuclear genes play dominant roles in regulating these traits, and further functional studies are required to clarify the extent to which chloroplast variation modulates tea quality traits.

Phylogenetic analysis based on 80 shared PCGs placed all six accessions firmly within the *Camellia* genus, revealing clear distinctions among the three genome types. Type 1 cultivars (‘Anjibaicha’ ‘Dahaocha’ ‘Fuyun 6hao’ and ‘Zhongcha 108’) clustered closely with *C. ptilophylla*, whereas Type 2 (‘Fuding Dabaicha’) and Type 3 (‘Dayecha’) exhibited affinities with *C. leptophylla* and *C. grandibracteata*, respectively. These relationships provide new insights into the genetic differentiation and domestication history of Hainan tea germplasm. The clear separation into three types likely reflects underlying chloroplast genome variation, including sequence divergence in coding and non-coding regions, which preserves signatures of ancestral lineages and historical hybridization events. The high bootstrap support (≥87) across most nodes attests to the robustness of the phylogenetic topology and underscores the power of chloroplast genomic data for resolving intrageneric evolutionary relationships [3,6].

The identification of the hypervariable intergenic spacer *trnT–psbD*, showing high nucleotide diversity, provides a promising candidate for DNA barcoding and molecular marker development. Within *Camellia*, analyses of *C. duntsa* and its close relatives revealed highly variable regions such as *ycf1* and *ndhF–rpl32*, which differ from *trnT–psbD*, indicating that the location of hypervariable regions can vary even among closely related species [38]. Comparisons with other genera, such as *Taxodium* (*clpP–accD* and *ycf1*) [9] and *Paraphalaenopsis* (*psbM–trnD* and psbB) [9] and *Paraphalaenopsis* (*psbM-trnD* and *psbB*) [39], further illustrate that highly variable regions differ across genera, underscoring the reliability of *trnT–psbD* as a genus-specific chloroplast marker. While this region appears suitable for species discrimination and phylogenetic studies, its utility as a diagnostic marker still requires broader validation across diverse populations. Notably, this study is limited by the relatively small number of individuals analyzed, and thus the present results should be regarded as preliminary. Consistent with patterns observed in many angiosperms, nucleotide diversity was higher in the LSC and SSC regions than in the IRs, reflecting the evolutionary conservatism of inverted repeat sequences [10]. Genome collinearity analysis confirmed the absence of major rearrangements, emphasizing the structural stability and evolutionary conservation of tea chloroplast genomes. Expanding future analyses to include additional wild and cultivated tea accessions from Hainan will deepen our understanding of local adaptation and accelerate marker-assisted breeding for desirable traits such as stress tolerance and leaf quality. Future analyses could combine chloroplast and nuclear SNP data and test the candidate marker across wild and cultivated Hainan populations to deepen understanding of local adaptation and facilitate marker-assisted breeding.

## 5. Conclusions

This study presents a comprehensive analysis of the complete chloroplast genomes of *C. sinensis* ‘hainanensis’ cultivars. We successfully assembled and annotated six *C. sinensis* accessions, revealing their typical structure and identifying three distinct genome types (Types 1, 2 and 3) based on structural variations. Comparative genomic analyses revealed conserved features, such as codon usage bias and repeat sequence patterns. Phylogenetic reconstruction placed the six accessions within the *Camellia* genus, elucidating their evolutionary relationships. We developed a polymorphic molecular marker based on a four-thymine insertion in the *trnT–psbD* intergenic spacer that can distinguish the Type 3 cultivar (‘Dayecha’). Our findings provide essential genomic resources and practical molecular tools for future studies on the evolution of the tea and the breeding of this valuable resource.

## Figures and Tables

**Table 1:** Assembly and structural features of six *Camellia* chloroplast genomes.

Species	Type	Cultivar	Region	Length (bp)	GC Content (%)
*C. sinensis* ‘hainanensis’	Type 1	Anji baicha	Total	157,025	37.30
			LSC	86,586	35.33
			IR-A	26,081	42.95
			SSC	18,277	30.55
			IR-B	26,081	42.95
	Type 1	Fuding dahaocha	Total	157,025	37.30
			LSC	86,586	35.33
			IR-A	26,081	42.95
			SSC	18,277	30.55
			IR-B	26,081	42.95
	Type 1	Fuyun 6	Total	157,025	37.30
			LSC	86,586	35.33
			IR-A	26,081	42.95
			SSC	18,277	30.55
			IR-B	26,081	42.95
	Type 1	Zhongcha 108	Total	157,025	37.30
			LSC	86,586	35.33
			IR-A	26,081	42.95
			SSC	18,277	30.55
			IR-B	26,081	42.95
	Type 2	Fuding dabaicha	Total	157,099	37.30
			LSC	86,643	35.32
			IR-A	26,090	42.95
			SSC	18,276	30.56
			IR-B	26,090	42.95
	Type 3	dayecha	Total	157,105	37.29
			LSC	86,632	35.32
			IR-A	26,094	42.94
			SSC	18,285	30.51
			IR-B	26,094	42.94

Note: Summary of the total length, structure, and GC content of the newly assembled chloroplast genomes from six tea cultivars.

**Table 2:** Gene composition of the *Camellia* chloroplast genomes.

Group of Genes	Name of Genes
Subunits of NADH-dehydrogenase	*ndhA*, *ndhB(×2)*, *ndhC*, *ndhD*, *ndhE*, *ndhF*, *ndhG*, *ndhH*, *ndhI*, *ndhJ*, *ndhK*
Subunits of photosystem Ⅰ	*psaA*, *psaB*, *psaC*, *psaI*, *psaJ*
Subunits of photosystem Ⅱ	*psbA*, *psbB*, *psbC*, *psbD*, *psbE*, *psbF*, *psbH*, *psbI*, *psbJ*, *psbK*, *psbL*, *psbM*, *psbN*, *psbT*, *psbZ*, *ycf3*
Subunits of cytochrome b/f complex	*petA*, *petB*, *petD*, *petG*, *petL*, *petN*
Subunits of ATP synthase	*atpA*, *atpB*, *atpE*, *atpF*, *atpH*, *atpI*
Large subunit of rubisco	*rbcL*
Small subunit of ribosome	*rps2*, *rps3*, *rps4*, *rps7(×2)*, *rps8*, *rps11*, *rps12(×2)*, *rps14*, *rps15*, *rps16*, *rps18*, *rps19*
Large subunit of ribosome	*rpl2(×2)*, *rpl14*, *rpl16*, *rpl20*, *rpl22*, *rpl23(×2)*, *rpl32*, *rpl33*, *rpl36*
DNA dependent RNA polymerase	*rpoA*, *rpoB*, *rpoC1*, *rpoC2*
rRNA genes	*rrn4.5S(×2)*, *rrn5S(×2)*, *rrn16S(×2)*, *rrn23S(×2)*
tRNA genes	*trnA-UGC(×2)*, *trnC-GCA*, *trnD-GUC*, *trnE-UUC*, *trnF-GAA*, *trnfM-CAU*, *trnG-UCC*, *trnH-GUG*, *trnI-CAU(×2)*, *trnI-GAU(×2)*, *trnK-UUU*, *trnL-CAA(×2)*, *trnL-UAA*, *trnL-UAG*, *trnM-CAU*, *trnN-GUU(×2)*, *trnP-UGG*, *trnQ-UUG*, *trnR-ACG(×2)*, *trnR-UCU*, *trnS-GCU*, *trnS-GGA*, *trnS-UGA*, *trnT-CGU*, *trnT-GGU*, *trnT-UGU*, *trnV-GAC(×2)*, *trnV-UAC*, *trnW-CCA*, *trnY-GUA*
Maturase	*matK*
c-type cytochrom synthesis gene	*ccsA*
Envelope membrane protein	*cemA*
Protease	*clpP*
Subunit of Acetyl-CoA-carboxylase	*accD*
Translational initiation factor	*infA*
Genes of unknown functions Open Reading	*ycf1(×2)*, *ycf2(×2)*, *ycf4*, *ycf15(×2)*

Note: Catalog of annotated genes within the chloroplast genomes, categorized by functional groups. Gene copy numbers are indicated in parentheses (e.g., ×2).

## Data Availability

The genome sequences are available in GenBank under accession numbers PX427667-PX427672.The sequencing reads are available under BioProject PRJNA1335661, BioSamples SAMN52018973-SAMN52018978, and SRA accessions SRR35651280-SRR35651285.

## Supplementary Materials

The following supporting information can be downloaded at: https://www.mdpi.com/article/10.3390/biology15010007/s1, Figure S1: Coverage depth of six *C. sinensis* ‘Hainanensis’ chloroplast genomes; Figure S2: Sequence conservation profiles across the three chloroplast genome types, using Type 1 as the reference; Table S1: Raw sequencing data quality statistics for the six Camellia accessions; Table S2: IR/SC junction positions, boundary-associated genes, and partial duplications in the three plastome types of Camellia ‘Hainanensis’; Table S3: Relative Synonymous Codon Usage (RSCU) of codons for each amino acid in the three type chloroplast genome; Table S4: SSRs identified in three type plastomes; Table S5: Tandem repeat sequences in the three type plastomes; Table S6: Dispersed repeat sequences in the three type plastomes; Table S7: List of species and their accession numbers used to construct the phylogenetic tree; Table S8: Nucleotide Diversity Analysis Results of the chloroplast genomes; Table S9: Primers for verifying InDels among tea varieties for barcodes development.

## Abbreviations

PCR	Polymerase chain reaction
SSR	Simple sequence repeat
ML	Maximum-likelihood
BI	Bayesian inferences;
NCBI	National Center for Biotechnology Information
BLAST	Basic Local Alignment Search Tool
PCGs	Protein-coding gene sequences
