# Supplementary material for: Chloroplast Genome Analysis of Six Camellia sinensis Accessions: Genetic Divergence, Adaptive Evolution, and Molecular Marker Development
Source: Biology (Basel). 2025 Dec 19;15(1):7. doi: 10.3390/biology15010007 (PMC12784810; doi:10.3390/biology15010007)
Supplement: Supplementary file 1 [file biology-15-00007-s001.zip › Figures S1-S2.pdf]

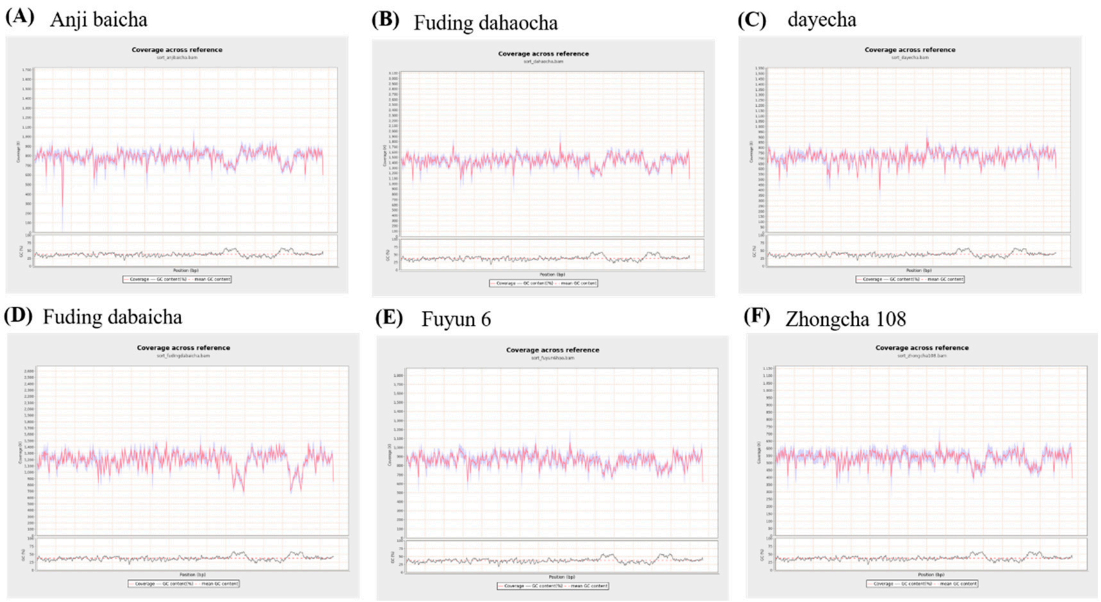

Figure S1 Coverage depth of six *C. sinensis* ‘Hainanensis’ chloroplast genomes.

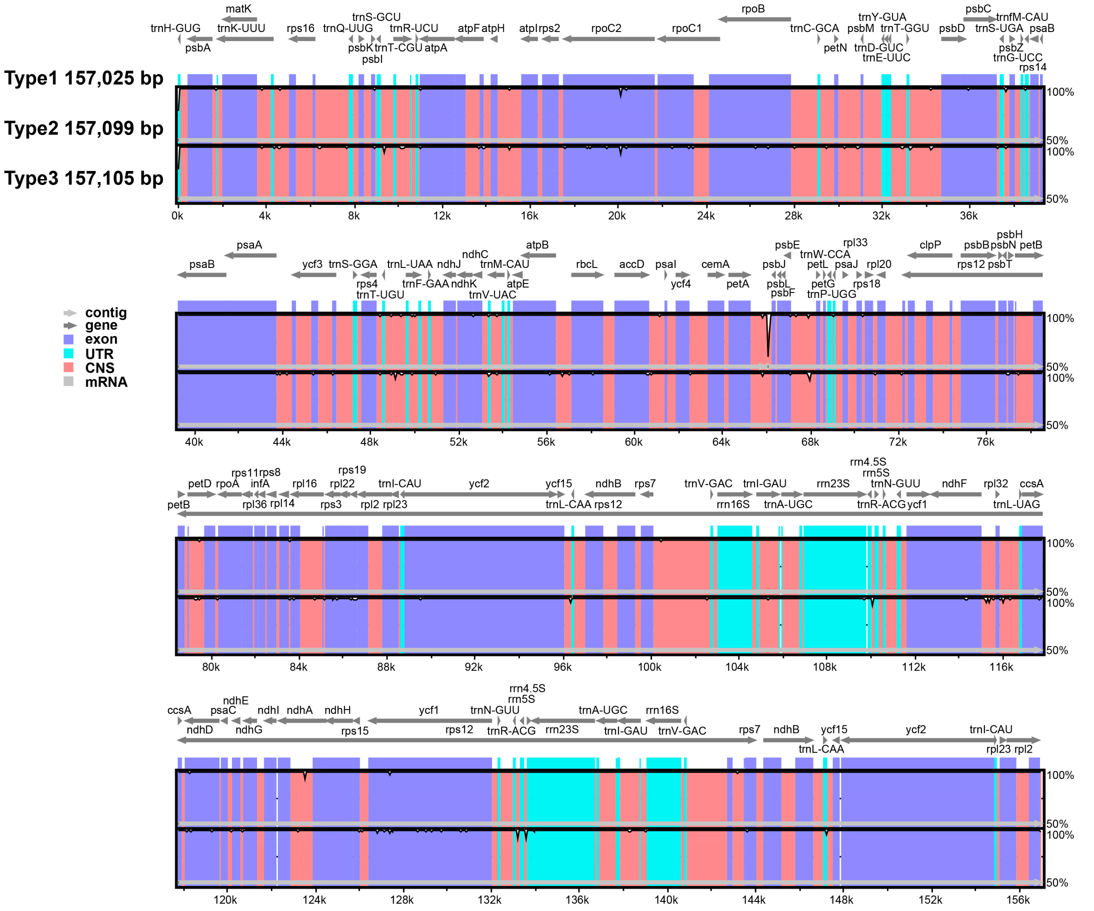

Figure S2. Sequence conservation profiles across the three chloroplast genome types, using Type 1 as the reference.
